# A Preliminary Report upon Forage-Poisoning of Horses (So-Called Cerebro-Spinal Meningitis)

**Published:** 1900-11

**Authors:** Leonard Pearson

**Affiliations:** Veterinary Department, University of Pennsylvania


### A PRELIMINARY REPORT UPON FORAGE-POISONING OF HORSES (SO-CALLED CEREBRO-SPINAL MENINGITIS).

By Leonard Pearson, B.S., V.M.D.,
VETERINARY DEPARTMENT, UNIVERSITY OF PENNSYLVANIA.

The disease of horses commonly known as cerebro-spinal meningitis has long been supposed to result from the ingestion of food that has undergone fermentation or putrefaction or has become mouldy. The evidence in favor of this view has, however, never been of a direct but only of a circumstantial nature. While the disease has in many instances occurred on farms and in stables where horses were fed on mouldy or musty grain or ground feed, damaged hay or spoiled ensilage, and it has been assumed that such foods produced the disease, there has always been a lack of proof, first, that these foods were poisonous, and, second, that some other influence had not produced the disease. This absence of proof is due to no lack of efforts to fix the responsibility on suspected foodstuffs. Experiments have been made in large numbers in which suspected materials have been fed to horses in the attempt to produce the disease called cerebro-spinal meningitis, but all of these trials have resulted negatively. So far as the literature of this subject shows, cerebro-spinal meningitis, so-called, has never been produced artificially or under experimental conditions.

Friedberger and Fröhner state (*Specielle Pathologie und Therapie*, 1896, fourth edition), in connection with the discussion of mould-poisoning, that feeding experiments are usually without result; but, they add, that it is illogical to conclude from this, in the face of the clinical evidence, that moulds do not have pathogenetic properties. They do not cite any experiments to show that mouldy foods are dangerous; all of their evidence appears to be clinical.

Case. On October 29, 1900, I was asked by Dr. Francis Bridge to see with him a stable in which five horses had died of cerebro-spinal meningitis, so-called. The purpose of the consultation was to determine if possible the origin of the disease.

It was found that the horse stable consisted of a row of seven stalls across one end of the stone basement of a large barn. One long side of the basement was against an embankment and had no windows. The other long side was protected by an overhang. There were windows under the overhang and in each end of the barn. In front of the row of horse stalls and running at right angles to it were two rows of cows with stalls for about forty animals. There were two silos on the embankment side of the stable. The silage was thrown down into a dark room, formerly used as a root-cellar, opening into the stable at about the middle. The partition between the part of the stable occupied by the cows and that occupied by the horses consisted of the front of the horses' mangers. This did not extend above the level of the feed-boxes.

A silo had been opened about one week before the first cases developed. The silage was somewhat mouldy on top and had a musty odor. This condition extended down around the sides for several feet. No silage was known to have been fed to the horses, although some could get on their hay, which remained for a time, after it was thrown down from the loft, in the passageway in front of the cows. It was also possible that some of the milkers may have fed a little silage to the horses.

The hay fed to the horses was of good quality and in good condition. The concentrated feed was a mixture of oats, corn, and bran, and appeared to be in good condition. It was kept in a covered feed bin in the passageway between the horse mangers and the cow stalls.

All of the seven horses in the stable became weak, showed muscular tremors, difficulty in chewing and swallowing, and gradually progressive paresis, which terminated in death in five instances. The other two horses were removed to another barn and recovered. The duration of the disease was from two to four days. No autopsies were made.

Suspecting the silage, on account of experience in previous outbreaks and on account of the very mouldy condition of some of it, I obtained a sample and took it to the Veterinary Hospital of the

University of Pennsylvania for trial. For the trial a nine-year old-gelding was used that had been in the hospital for two months. This gelding had been quite stiff from osteoporosis, but had recovered largely from his lameness, was well nourished, vigorous, and in good general condition.

The feeding experiment started on October 30th and continued until November 2d. The horse ate altogether approximately one-half bushel of silage mixed with oats and bran. November 2d he ate and swallowed slowly and with some difficulty. His temperature was 100° F. There was no evidence of pain. November 3d there was well-marked paresis of the throat and of the muscles of mastication. The temperature was 100.5° F. There was twitching of the muscles of the flank and shoulder, desire to lie down much of the time, and some difficulty in arising. The inability to swallow continued, and the general muscular weakness progressed. In the evening the horse was unable to stand. The brain was clear. There was no pain. The horse died at 9 A.M., November 4th. Autopsy negative except for swelling and dark red, almost black color of mucous membrane of pharynx and glottis. The mucous membrane of the larynx was congested. The mucous membrane of the stomach was also much congested and showed some ecchymotic spots on soft mucosa. The stomach contents had a putrid odor. A bolus of partially chewed hay was lodged between the teeth and cheek, and this had a very putrid odor. There appeared to be an excess of cerebro-spinal fluid. The brain and cord and their meninges were normal.

Another horse, a gelding ten years old, with ringbones on both front pasterns, but otherwise healthy, was given on November 5th four gallons of water that had percolated through a bushel of silage. November 6th he was given three gallons of water from the same silage. November 8th he was given six quarts of silage. November 9th he was offered four quarts of silage, but did not eat more than half of it. Up to November 9th in the afternoon no abnormal condition was noted. It was then observed that he chewed and swallowed slowly. In the evening there was a little tremor of the muscles of the shoulder and partial paralysis of the throat, but he could drink very slowly. He laid down most of the time and was weak. Temperature 98.2° F. No pain. November 10th the horse was found dead in his stall, having died during the night. Upon autopsy lesions similar to those described above were found, but all were less well marked. Other investigations in this connection are now being made, and will be reported later.

As to the name that is usually applied to this disease—*i. e.*, cerebro-spinal meningitis—it is evident that the name is inappropriate, because there is no evidence of inflammation in the meninges of the brain or cord. Since this disease closely resembles the sausage-poisonings and meat-poisonings of man and the carnivora, and since the observations recorded above show that the as yet undiscovered infectious or toxic principle resides in the food, I wish to suggest the name "forage-poisoning" as one that would be descriptive and accurate.

